# The Practice of Obstetrics

**Published:** 1901-12

**Authors:** 


THE PRACTICE OF OBSTETRICS.—By American authors. Edited by Charles Jewett, M. D., Professor of Obstetrics and Gynecology in Long Island College Hospital, Brooklyn, N. Y. New (2nd) edition, revised and enlarged. In one handsome octavo volume of 775 pages, with 445 engravings in colors and black, and 35 full-page colored plates. Cloth, net, $5.00; leather, net, $6.00; half morocco, net, $6.50. Lea Brothers & Co., Publishers, Philadelphia and New York.

Dr. Jewett's excellent work presents the best teaching on the present day obstetrics untrammeled by out-of-date theories and methods. The language is clear and uninvolved.

The author's collaborators are all men well known to the profession, most of them being connected with medical colleges and hospital clinics as teachers and demonstrators.

The illustrations contained in the present edition deserve special mention. All of them are excellently done, while most of the colored plates are strikingly rich and beautiful. Some illustrate well conditions and operations which are usually not clearly shown in even the most expensive and pretentious treatises.

Dr. Jewett deserves to be congratulated upon the results of his faithful and painstaking work, which certainly attests his broad experience as a teacher, author and editor. With such a text-book at hand, the obstetrician can feel himself well equipped for any emergency. R.

